# Factors affecting spiritual care competency of mental health nurses: a questionnaire-based cross-sectional study

**DOI:** 10.1186/s12912-023-01302-z

**Published:** 2023-06-13

**Authors:** Kuei-Hsiang Han, Kuo-Chuan Hung, Yu-Shian Cheng, Weilun Chung, Cheuk-Kwan Sun, Chia-Chan Kao

**Affiliations:** 1 Department of Psychiatry, Tsyr-Huey Mental Hospital, Kaohsiung Jen-Ai’s Home, Taiwan; 2 Department of Post Baccalaureate Nursing and Department of Nursing, I-Shou University, Medical Campus, No. 8, Yida Rd., Jiaosu Village, Yanchao District, Kaohsiung City, 82445 Taiwan; 3 Department of Anesthesiology, Chi Mei Medical Center, Tainan City, Taiwan; 4 Department of Emergency Medicine, E-Da Hospital, I-Shou University, Kaohsiung, Taiwan; 5 School of Medicine for International Students, College of Medicine, I-Shou University, Kaohsiung, Taiwan; 6 Department of Medical Research E-Da Hospital, I-Shou University, Kaohsiung, Taiwan

**Keywords:** Spiritual care competency, Mental health nurses, Personality traits, Professional development, Education

## Abstract

**Background:**

Although providing spiritual care is an important part of holistic nursing care for psychiatric patients, factors associated with spiritual care competency in mental health nurses remain unclear. The aim of our study was to explore a possible association of personal and external factors with spiritual care competency in mental health nurses.

**Methods:**

This prospective questionnaire-based cross-sectional study was conducted by inviting mental health nurses from mental health hospitals and tertiary referral centers. Personality traits and spiritual care competency were assessed by using [1] “big-five Mini-Markers” questionnaire, and [2] spiritual care competency scale, respectively. From the 250 mental health nurses being invited, 239 valid questionnaires were valid for final analysis. Statistical analyses including descriptive statistics, ANOVAs, t-tests, and hierarchical multiple regression models were used to investigate the associations between personal/external factors and their spiritual care competency in mental health nurses.

**Results:**

The mean age of the 239 participants was 35.96 ± 8.11 and the mean years of working experience was 9.41 ± 7.06. Over 90% of them had no experience of providing spiritual care. There were significant positive correlations of spiritual care competency with the experience of delivering spiritual care (*p* < 0.001), previous participation in spiritual care education programs (*p* = 0.045), a longer working experience (*p* = 0.014), and a higher education level (postgraduate vs. college, *p* = 0.006), as well as the personality components of “Conscientiousness” (*p* < 0.001), “Agreeableness” (*p* < 0.001), “Extraversion” (*p* = 0.03), and “Openness/Intellect” (*p* < 0.001).

**Conclusions:**

Both personal and external factors may be related to the self-perception of spiritual care competency among mental health nurses. These findings may help mental health nurses understand the possible positive and negative associations of their personality components with their spiritual care abilities. Moreover, our identification of the positive impacts of educational programs and previous experience of spiritual care on spiritual care competency may underscore the importance of tailoring appropriate training programs to cater for the individual needs of mental health nurses.

## Introduction

Spiritual care, which is accepted as an indispensable part of holistic nursing care [1], involves the assessment and individualized planning of one’s spiritual/religious needs with the aim of helping patients restore a balance between the physical, psychosocial, and spiritual aspects of self [2]. While the meaning of spirituality is multidimensional and can vary widely at cultural and individual levels [3, 4], most healthcare researchers have focused spiritual care on the experiential and existential aspects of life that may include but are not restricted to religious belief, values, meaning of life, a sense of connectedness and belonging to important others, as well as wider society and higher powers [5–10]. Therefore, addressing spiritual needs in nursing care involves not only appropriate communication between nursing staff and patients [11, 12], but also multidisciplinary collaboration of the entire medical team [13] and the participation of family members in order to deliver successful holistic nursing care [14].

While provision of spiritual care may diminish the negative impacts of physical illnesses on the patients’ self-image [15, 16], individuals with mental health issues often have a greater need for spiritual support than those afflicted with other medical illnesses [17]. Indeed, not only are psychiatric patients more open to discussion about spirituality [18], but those with major mental illnesses are also more likely to be troubled by spiritual issues compared to those without [19]. Moreover, discussion about spirituality can be important for improving the quality of communication between mental health workers and psychiatric patients [20]. In concert with this finding, failure to provide spiritual care could have negative impacts on both patients and health workers [21, 22]. Several observational studies and meta-analyses also showed that a significant proportion of individuals diagnosed with mental illnesses regarded spirituality or religion as an essential resource for coping with difficult or stressful life events in the process of recovery [23, 24]. Furthermore, the importance of addressing spiritual issues in psychiatric patients was reflected by the results of a study showing that paying attention to spiritual issues in conversation with patients could maximize therapeutic benefit from their relationship with mental health professionals [25].

However, previous studies have reported a wide discrepancy between the realization of the need for spiritual care and actual practice [1], in which nursing staff are often uncertain about how they could deliver spiritual care to patients diagnosed with mental health problems [26]. Another study further found that a significant portion of mental health workers were unaware of their patients’ spiritual needs, although religion was considered an important aspect of life in most patients with mental illnesses [27]. Therefore, in order to raise awareness and also improve the competency of providing spiritual care for psychiatric patients, it is important to identify potential factors that are associated with mental health nurses’ ability to provide spiritual care [9].

Prior investigations have revealed a correlation of personal factors such as age, personal values, and previous work experience with spiritual care competency in nurses [28–31]. On the other hand, external factors such as the policy of institutions and provision of educational programs were also found to be essential for enhancing nurses’ spiritual care abilities [28–31]. Nevertheless, given the importance of providing spiritual care for psychiatric patients [17], it is surprising that the associations of personal and external factors with spiritual care competency of mental health nurses have received little attention in the past [32]. Another observational study comparing the self-rated competence towards spiritual care between nurses of various subspecialties showed that spiritual nursing care competency and its link to other factors may vary in different health sectors [33]. Moreover, despite the findings that personality traits are associated with the provision of spiritual care nursing and patients’ satisfaction [34, 35], none of the studies focused on mental health nurses. Overall, there was limited research on factors that may be related to spiritual care competency in mental health nurse [32].

Therefore, to gain an insight into the correlations of different personal and/or external factors with spiritual care competency of mental health nurses, this cross-sectional questionnaire-based study aimed at [1] investigating the relationship between spiritual care competency and personal factors such as demographic and personality-related factors, and [2] evaluating the associations of external factors especially education and training with competency of spiritual care in mental health nurses.

## Methods

### Study design and participants

The current prospective questionnaire-based cross-sectional study was conducted from April 1 to July 31, 2017 on registered nurses with age over 20 who had a valid license and worked as first-line care providers for inpatients during the study period in the psychiatry departments of either mental health hospitals or tertiary referral centers with acute and chronic psychiatric wards. We excluded those who did not participate in primary patient care and those whose clinical practice lasted less than three months.

### Data acquisition

Based on geographical accessibility, we communicated with the head nurses of all tertiary referral centers and mental health hospitals (five and eight, respectively) in three major cities, through electronic mails to determine their willingness to participate in the present study. Three of the five tertiary referral centers and five of the eight mental health hospitals agreed to take part in the current study. The head nurses who acquiesced to the research program then collected relevant information from their nursing staff who agreed to join voluntarily. One investigator (K,-H. H) was responsible for providing a full explanation for the participants regarding the aims and procedures of the present investigation, ensuring the confidentiality of the acquired information, as well as distributing and collecting the questionnaires in person. The average time for completion of the questionnaires ranged from 20 to 40 min.

The sociodemographic parameters of the current study, namely personal characteristics including age (i.e., 21–30, 30–40, > 40), marital status, education level (i.e., technical institute, university graduate, postgraduate), religious belief, working experience (i.e., < 5 years, 5–10 years, > 10 years), and previous participation in educational programs on holistic/spiritual care (yes/no) are summarized in Table 1.

**Table 1:** Associations of study participant characteristics with spiritual care competency (N = 239)

Parameters	Group	N	%	Mean ± SD	Spiritual care competency
Age (years)	21–30	63	26.4	35.96 ± 8.11	
	30–40	114	47.7		
	≥ 40	62	25.9		
Working experience (years)	< 5	84	35.1	9.41 ± 7.06	
	5–10	51	21.3		
	≥ 10	104	43.6		
Medical institute	Mental health hospital	149	62.3		44.70 ± 7.74
	Tertiary referral hospitals	90	37.7		45.32 ± 7.92
Job title ranking	Junior	120	50.2		44.70 ± 7.87
	Middle	82	34.3		44.07 ± 6.70
	Senior	37	15.5		47.62 ± 9.30
Education level	Occupational institutes	65	27.2		44.05 ± 7.63
	Universities	150	62.8		44.63 ± 7.51
	Postgraduate schools	24	10.0		49.29 ± 8.86 (3 > 1*, 3 > 2*)
Marital status	Married	113	47.3		46.17 ± 8.13
	Divorced	15	6.3		44.27 ± 7.20
	Single	111	46.4		43.77 ± 7.38
Religion	No	61	25.5		44.20 ± 8.16
	Buddhism and Taoism	156	65.3		45.33 ± 7.72
	Western religions	22	9.2		44.23 ± 7.46
Continuing education on spiritual care	Yes	37	15.5		47.30 ± 7.58*
	No	202	84.5		44.51 ± 7.77
Continuing education on palliative care	Yes	68	28.5		46.46 ± 7.43
	No	171	71.5		44.33 ± 7.87
Continuing education on spiritual nursing care	Yes	31	13.0		46.55 ± 6.49
	No	208	87.0		44.70 ± 7.95
Participation in course on spiritual growth	Yes	27	11.3		45.19 ± 8.32
	No	212	88.7		44.91 ± 7.74
Experience of providing spiritual care	Yes	23	9.6		51.04 ± 6.50**
	No	216	90.4		44.29 ± 7.65
Agreeableness (A)	Score: 1–34	127			40.32 ± 7.42
	Score: 35–56	112			47.91 ± 7.14**
Conscientiousness (C)	Score: 1–34	125			42.57 ± 7.55
	Score: 35–56	114			47.96 ± 7.04**
Openness/Intellect (O)	Score: 1–34	123			42.53 ± 7.29
	Score: 35–56	116			47.58 ± 7.49**
Extraversion (E)	Score: 1–40	134			43.91 ± 7.49
	Score: 41–56	105			46.10 ± 7.99*
Emotional Stability (E)	Score: 1–40	129			44.15 ± 7.91
	Score: 41–56	110			45.77 ± 7.62

N: number of participants; SD: standard deviation; *p < 0.05; **p < 0.01

There were two questionnaires for the current investigation, one of which (i.e., the international “big-five Mini-Markers” questionnaire [36]) identified the personality components of the participants. The questionnaire consisted of five main subscales, including “Agreeableness”, “Conscientiousness”, “Openness/Intellect”, “Extraversion”, and “Emotional Stability” (Table 2). There were eight question items for each subscale, giving a total of 40 items. The participant then assigned to each item a score from 1 to 7 that denoted “very incompatible”, “incompatible”, “a bit incompatible”, “neutral”, “a bit compatible”, “compatible”, and “very compatible”, respectively. It is one of the most frequently used culturally generalizable tools [37] to assess the correlation of personality components with spirituality due to its ability to investigate the trait-like characteristics that affect people’s perception of the world as well as their thoughts and behavior [38, 39]. The current study adopted the Chinese version of the questionnaire that has been validated and widely utilized in previous investigations [40, 41].

**Table 2:** Personality components of study participants based on International English Big-Five Mini-Markers (N = 239)

Personality component	mean	SD	median
**Agreeableness (A)**	**43.25**	**7.45**	**44**
Rude	5.64	1.23	
Sympathetic	5.54	1.25	
Harsh	5.50	1.27	
Inconsiderate	5.46	1.30	
Unkind	5.41	1.27	
Kind	5.27	1.24	
Cooperative	5.24	1.13	
Warm	5.20	1.25	
**Conscientiousness (C)**	**39.96**	**7.15**	**40**
Untidy	5.33	1.20	
Neat	5.23	1.28	
Inefficient	5.13	1.31	
Organised	4.91	1.14	
Careless	4.87	1.22	
Systematic	4.86	1.15	
Disorganised	4.85	1.16	
Efficient	4.79	1.14	
**Openness/Intellect (O)**	**34.82**	**6.82**	**34**
Intellectual	4.54	1.09	
Artistic	4.51	1.20	
Intelligent	4.46	1.08	
Unimaginative	4.42	1.24	
Uncreative	4.35	1.25	
Creative	4.27	1.30	
Deep	4.27	1.12	
Philosophical	4.00	1.18	
**Extraversion (E)**	**34.36**	**7.52**	**34**
Energetic	4.85	1.19	
Talkative	4.73	1.31	
Quiet	4.49	1.44	
Outgoing	4.47	1.31	
Untalkative	4.33	1.39	
Extroverted	4.32	1.34	
Shy	3.65	1.40	
Reserved	3.52	1.18	
**Emotional Stability (E)**	**30.37**	**5.83**	**30**
Envious	4.37	1.37	
Jealous	4.05	1.43	
Unenvious	3.85	1.32	
Emotional	3.85	1.38	
Unanxious	3.65	1.17	
Moody	3.66	1.42	
Unworried	3.55	1.30	
Anxious	3.38	1.31	

N: number of participants; SD: standard deviation

The second questionnaire that assessed spiritual care competency was first described by van Leeuwen et al. (2009) [42]. The original spiritual care competency scale included six categories and a total of 27 items [42]. For the current study, we modified the questionnaire and tested its validity by using the content validity index [43]. Four senior professionals specialized in psychiatric nursing were invited to modify the questionnaires according to their suitability in terms of cultural and linguistic backgrounds. The final questionnaire comprised 13 items in three main categories, including the quality of spiritual care (seven items), psychological support and referral (four items), and communication (two items) (Table 3). Each item was rated with a five-point Likert scale (1: strongly disagree, 2: disagree, 3: neutral, 4: agree, 5: strongly agree), giving a total score ranging from 13 to 65. A higher score indicated a better spiritual care competency. We used factor analysis to evaluate the construct validity of the questionnaires, while we adopted average inter-item correlation and Cronbach’s α to examine their internal consistency. The three subscales of the questionnaire for assessing spiritual care competency were “quality of spiritual care” (seven items), “psychological support and referral” (four items), and “communication” (two items) with Cronbach’s α values of 0.92, 0.84, and 0.93, respectively, and an overall value of 0.92. With regard to the “big-five Mini-Markers” questionnaire, the Cronbach’s α values for the subscales “Agreeableness”, “Conscientiousness”, “Openness/Intellect”, “Extraversion”, and “Emotional Stability” were 0.89, 0.89, 0.87, 0.86, and 0.66, respectively, giving an overall value of 0.91.

**Table 3:** Spiritual care competency of study participants (N = 239)

Component of Assessment	mean	SD
**Overall spiritual care abilities**	**44.94**	**7.79**
**Communication** (two items)	**4.18**	**0.77**
Showing respectful attitudes towards patient’s spirituality	4.20	0.81
Listening carefully to patient’s ‘life story’	4.16	0.73
**Psychological support and referral** (four items)	**3.39**	**0.87**
Assisting patients with their participation in spiritual activities (e.g., worshiping, praying)	3.52	0.84
Providing patients with objects necessary for spiritual activities (e.g., Bible, audiovisual materials)	3.50	0.85
Collaborating with medical team when delivering spiritual care	3.36	0.86
Referring patients with spiritual needs to religious teacher if necessary	3.20	0.93
**Qualities of spiritual care** (seven items)	**3.29**	**0.82**
Tailoring a spiritual care plan with medical team to meet patients’ spiritual requirements	3.44	0.87
Having ability to evaluate patients’ difficulties in expressing their spiritual needs	3.42	0.85
Being capable of assessing patients’ spiritual needs	3.38	0.75
Being willing to discuss with patients about the need for a spiritual care plan	3.31	0.84
Being capable of discussing with patients’ families about spiritual care planning	3.18	0.79
Being able to help patients cope with spiritual crisis (e.g., disease-related challenge)	3.16	0.81
Having ability to formulate a spiritual nursing care plan for patients in need	3.11	0.85

N: number of participants; SD: standard deviation

### Data analysis

To produce statistically meaningful results, we used linear multiple regression analysis in the G*power software (3.1.0) to estimate the required sample size by setting an expected effect size, an alpha level, and a power at 0.15, 0.05, and 0.95, respectively [44]. Focusing on the key outcome variable of spiritual care competency, a total sample size of 189 individuals would be needed. Analytic strategies of the current study included descriptive statistics, ANOVAs, t-tests, and hierarchical multiple regression models. Based on the personal characteristics of the participants (e.g., age) and scores on different personality components (i.e., 1–34 vs. >35), we divided the participants into different groups. Detailed information is provided in Table 1. We then used t-tests to determine the significance of difference between two groups and ANOVA for comparing the differences for three or more groups. Dummy coding was used for regression analysis of categorical variables, while hierarchical multiple regression was adopted to examine the correlations between multiple variables. All statistical analyses were conducted with the SPSS software (version 22.0). Mean values are presented as mean ± standard deviation (SD). A probability value (*p*) less than 0.05 was considered statistically significant.

### Research ethics

The protocol of the present study was approved by the Institutional Review Board (IRB) of our institute (IRB number: EMRP-105-120) and all procedures were conducted in compliance with the Declaration of Helsinki. All participants needed to sign an informed consent that contained detail regarding the purpose and procedures of the research. As an incentive, a tableware gift was given to participants who completed the questionnaires.

## Results

### Sample description

Of a total of 250 questionnaires sent to the participants, up to 97.6% (i.e., 244) were retrieved. After removal of five questionnaires deemed ineligible because of missing items, data from 239 questionnaires (i.e., over the required minimum of 189) were acquired for analysis. The average age of the participants was 35.96 ± 8.11 with most of them being in the range between 30 and 40 (47.7%, n = 114) (Table 1). Their average working experience was 9.41 ± 7.06 years with most participants working for more than 10 years (43.6%, n = 104). Over half were working in mental health hospitals (62.3%, n = 149) and about half of all participants (50.2%, n = 120) belonged to a junior rank (Table 1). Most were university graduates (62.8%, n = 150). The population of participants who were married at the time of the survey (47.3%, n = 113) was slightly larger than that of those who were single (46.4%, n = 111), followed by divorcees (6.3%, n = 15). In terms of religious belief, more than half were Buddhists and Taoists (n = 156; 65.3%). Most participants had no previous experience of attending any continuing education program on spiritual or palliative care (84.5%, n = 202 and 71.5%, n = 171, respectively). Besides, the majority did not participate in continuing education course on spiritual nursing (87.0%, n = 208) or spiritual growth (88.7%, n = 212). Moreover, 90.4% of them reported no experience with spiritual care.

### Personality traits and spiritual care competency

In respect of personality scoring, the participants got a highest score on the subscale of “Agreeableness” (43.25 ± 7.45) but scored lowest on “Emotional Stability” (30.37 ± 5.83) (Table 2). Regarding scoring on spiritual care competency, their average score was 44.94 ± 7.79 (Table 3).

### The associations of personal characteristics and external factors with spiritual care competency

Statistical analyses using t-test and ANOVA (Table 1) showed a correlation of the differences in overall competency in spiritual care with the psychiatric nurses’ education level (*p* = 0.013), previous participation in spiritual care education programs (*p* = 0.045), and experience of delivering spiritual care (*p* < 0.001). Regarding the effect of personality factors on spiritual care competency, t-test analysis (Table 1) demonstrated a significantly better mean competency score among high scorers on “Extraversion”, “Openness/Intellect”, “Conscientiousness”, and “Agreeableness” than that of the low scorers (*p* = 0.031, *p* < 0.001, *p* < 0.001, and *p* < 0.001, respectively).

### Multiple regression analysis

Multiple regression analysis revealed significant positive correlations of spiritual care competency with the experience of delivering spiritual care (*p* < 0.001), previous participation in spiritual care education programs (*p* = 0.045), a longer working experience (*p* = 0.014), and a higher education level (postgraduate vs. college, *p* = 0.006), as well as the personality components of “Conscientiousness” (*p* < 0.001), “Agreeableness” (*p* < 0.001), “Extraversion” (*p* = 0.03), and “Openness/Intellect” (*p* < 0.001) (Table 4).

**Table 4:** *Correlations between demographic/personality factors and spiritual care competency on multiple regression analysis (N = 239)*

Demographic/personality factors	β	*p-*value	R²
Age Experience of delivering spiritual care	0.11 0.26	0.09 < 0.001	0.012 0.66
A longer working experience	0.16	0.014	0.025
a higher education level (postgraduate vs. technical institute)	0.18	0.006	0.036
previous participation in education on spiritual care program	0.13	0.045	0.017
previous participation in education on spiritual care program			
Agreeableness	0.35	< 0.001	0.125
Conscientiousness	0.34	< 0.001	0.119
Openness/Intellect	0.32	< 0.001	0.105
Extraversion	0.14	0.030	0.020

β: Beta coefficient; R²: R-squared

## Discussion

To our best knowledge, the current study is the first to examine the relationship between personality traits and spiritual care competency among mental health nurses. We also explored the relationship between spiritual care competency and demographic data as well as other external factors such as educational programs in mental health nurses that received little attention in the past. Our study found a positive association of spiritual competency with certain personal factors including a higher education level (postgraduate vs. technical institute), a longer working experience, as well as the personality components of “Agreeableness”, “Conscientiousness”, “Openness/Intellect”, and “Extraversion”. On the other hand, our results also showed that some external factors including previous participation in spiritual care education programs and the experience of delivering spiritual care were related to better spiritual care competency in mental health nurses. Although our results were similar to those of most previous studies [28–31, 35], our investigation provided additional information that particularly focused on mental health nurses. Therefore, the findings of our study may serve as an important reference for mental health nurses to understand their personal weaknesses and strengths in providing spiritual care as well as for health care institutions to organize appropriate training programs taking into consideration the roles of spiritual care experience and education in fostering such as key competency.

With regard to comparing our results with those of previous studies, the present investigation showed a mean overall item score of 3.46 ± 0.60 out of a full score of 5 (i.e., percentage score: 69.20%), which was similar to that of a number of previous studies [28–31, 33, 35, 45] that reported a percentage score ranging from 70% [45] to 77.3% [30]. While the previous studies covered a wide range of ethnic backgrounds and nurses from different health sectors [28–31, 33, 35, 45], our results were closest to those of a study with the same ethnic background that reported a score of 70% despite its focusing on nurses of different subspecialties [45].

The current study, which also investigated the relationships between demographic data and spiritual care competency, showed that postgraduate education and a longer working experience were linked to better spiritual care competency. The finding of a positive association between better spiritual care competency and a longer work experience was highly consistent with that in most previous studies [28, 30, 31], while postgraduate education was found to positively correlate with spiritual care competency in one report [28] but not investigated in the other two studies [30, 31]. this may suggest that work experience may be an important universal factor associated with spiritual care competency of nurses. On the other hand, because the participants of the previous study that also reported a positive correlation between postgraduate education and spiritual care competency [28] were from the same ethnic background as the current study, further investigations into the ethnic impact are warranted.

One important finding of the current study was the relationship between personality traits and spiritual care behaviors. There was only one previous study focusing on this particular topic [35]. Similar to our finding of an association between better spiritual care competency and four personality traits, namely “Extraversion”, “Openness/Intellect”, “Conscientiousness”, and “Agreeableness”, that study also demonstrated that these four personality traits were linked to more positive spiritual caring behaviors in hospital nurses in Greece [35]. Therefore, our findings and those of that study [35] suggested that these associations may be independent of health sectors and ethnic backgrounds. Besides nursing, a previous meta-analysis focusing on the association between religiosity and personality consistently demonstrated that “Openness/Intellect”, “Conscientiousness”, and “Agreeableness” correlated with more open and mature spirituality [46]. That study further showed that more open and mature spirituality was linked to a higher emotional stability and better mental health [46]. Taking into account the potential association of personality traits with spiritual care competency, our findings not only may help mental health nurses identify personal weaknesses and strengths but also highlight the potential benefits of implementing tailored education and training programs to reinforce spiritual care training. Moreover, given the dearth of research on the correlation between personality components and spiritual care competency among nursing staff [35], the finding of the present study could serve as an important reference and encourage further studies to explore the relationship between personality characteristics and spiritual care nursing.

Finally, the associations of educational programs and experience of delivering spiritual care with spiritual care competency were more extensively studied and consistently found to correlate with better spiritual care competency in previous studies [28, 29, 31, 35] and also in our investigation. These results are especially crucial for reinforcing favorable modifiable factors for spiritual care delivery among mental health care nurses through providing appropriate educational and training programs. Moreover, the finding of a positive association between previous experiences with spiritual care and spiritual care competency suggested that, in addition to enhancing the theoretical aspect of spiritual care nursing, hands-on practice may be equally important to enhance spiritual care competency.

## Limitations

There were several limitations in the present study; first, because cultural factors may have a strong influence on the interpretation of spirituality and also on the perception of spiritual caring behaviors [47], our results may not be extrapolated to mental health nurses from different cultural backgrounds. On the other hand, our findings of an association of better spiritual care competency with a longer work experience [28, 30, 31], and experience of spiritual care practice, as well as previous participation in relevant educational programs [28, 29, 31, 35] were highly consistent with those of previous studies, suggesting a universality of the correlations. Second, the cross-sectional nature of the study design could only provide information about the correlation between different factors and spiritual care competency rather than establish a causal relationship. Third, although the quantitative design of the current study helped to test the hypotheses of the associations of different factors with spiritual care competency in mental health nurses, it could not provide detail about individual perceptions of spiritual care as in qualitative research. Finally, while the correlations between demographic data and external factors such as education and previous training were also observed in many previous studies [28, 29, 31, 35], we could only find one study that also investigated the relationship between personality traits and spiritual care behaviors in nurses [35]. Although the results of that study were similar to ours, further studies are needed to verify the findings because of their preliminary nature.

## Conclusion

Our results demonstrated that certain personal factors including a longer work experience, higher education level, as well as certain personality traits including “Openness/Intellect”, “Conscientiousness”, and “Agreeableness” were associated with better spiritual care competency in mental health nurses. While the findings may help mental health nurses understand possible personal strengths and weaknesses towards their spiritual care abilities, our identification of the positive impacts of educational programs and previous experience of spiritual care on spiritual care competency may underscore the importance of tailoring appropriate training programs to cater for the individual needs of mental health nurses.

## Data Availability

Data cannot be shared based on the confidential agreement between the study participants and research team based on our protocol approved by the Institutional Review Board of our university. The data that support the findings of this study are available only on request from the first author (Name: Kuei-Hsiang Han, Email: yoyo03192000@yahoo.com.tw).

## Abbreviations

IRB: Institutional Review Board
*p*: probability value
SD: standard deviation
